# Effects of 6-Shogaol on Glucose Uptake and Intestinal Barrier Integrity in Caco-2 Cells

**DOI:** 10.3390/foods12030503

**Published:** 2023-01-21

**Authors:** Wenya Jiao, Yaxin Sang, Xianghong Wang, Shuo Wang

**Affiliations:** 1 College of Food Science and Technology, Hebei Agricultural University, Baoding 071000, China; 2 Tianjin Key Laboratory of Food Science and Health, School of Medicine, Nankai University, Tianjin 300071, China

**Keywords:** 6-shogaol, Caco-2 cells, glucose uptake, barrier function

## Abstract

As the main bioactive component in dried ginger, 6-shogaol has potential hypoglycemic activity, but its mechanism is still unclear. The process of carbohydrate digestion and glucose absorption is closely related to the enzymatic activity of epithelial brush cells, expression of glucose transporters, and permeability of intestinal epithelial cells. Therefore, this study explored the hypoglycemic mechanism of 6-shogaol from the perspective of glucose uptake, absorption transport, and protection of intestinal barrier function. Based on molecular docking, the binding energy of 6-shogaol and α-glucosidase is −6.24 kcal/mol, showing a high binding affinity. Moreover, a-glucosidase enzymatic activity was reduced (−78.96%) when the 6-shogaol concentration was 500 µg/mL. After 6-shogaol intervention, the glucose uptake was reduced; the relative expression of glucose transporters GLUT2 and SGLT1 were down regulated; and tight junction proteins ZO-1, Occludin and Claudin were up regulated in differentiated Caco-2 cells. This study confirmed that 6-shogaol effectively inhibits the activity of α-glucosidase and has beneficial effects on glucose uptake, protection of intestinal barrier function, and promotion of intestinal material absorption.

## 1. Introduction

The dried ginger (*Rhizoma zingiberis*) is derived from the dried rhizome of *Zingiber officinale Rose*. It has been reported that ginger has a variety of health benefits, such as antioxidant [1,2], anti-inflammatory [3], immune regulation [4], antiemetic [5], hypoglycemic [6,7], and lipid-lowering [8] properties, among others. Gingerols, as the main flavor and active components in ginger, are important contributors to the functional properties of ginger. The 6-shogaol belongs to a kind of gingerols, which has a higher concentration in dried ginger than in undried ginger [3]. This is because 6-gingerol is easily dehydrated and transformed into 6-shogaol under the influence of temperature during drying [9]. In recent years, studies have reported the hypoglycemic effect of 6-shogaol, including reducing blood sugar [10], improving kidney damage caused by diabetes [11], and diabetes cardiomyopathy [12]. For streptozotocin-induced diabetes mice, the administration of 6-shogaol significantly reduced blood glucose; improved insulin resistance; and alleviated pancreatic, kidney, and liver damage [10,11]. Based on the cell model of diabetes cardiomyopathy induced by high glucose, 6-shogaol pretreatment significantly increased its cell viability and reduced its apoptosis, reflecting the potential protective effect of 6-shogaol on the induction of diabetes cardiomyopathy [12]. In addition, it was found that 6-shogaol could effectively regulate the glucose utilization of 3T3-L1 adipocytes and C2C12 myotube cell lines [13]. These results indicate that 6-shogaol can be used as a therapeutic agent to prevent complications in patients with diabetes.

Type 2 diabetes (T2DM) is an endocrine disorder of protein, lipid, and carbohydrate metabolism, which is usually accompanied by a variety of complications [14]. Its main feature is the increase in fasting and postprandial blood glucose levels. At present, in addition to diet management, some chemosynthetic drugs are also often used to treat T2DM. However, it still has adverse reactions, such as hypoglycemia and gastrointestinal irritation [15]. Therefore, people pay more and more attention to reducing blood glucose and improving T2DM through active ingredients in food and drug homologous foods. In recent years, the relevant research on the hypoglycemic effect of natural active ingredients mainly focuses on the following aspects: (1) inhibiting the activity of related enzymes; (2) improving insulin resistance; (3) liver glycogen decomposition and gluconeogenesis. α-glucosidase plays a key role in improving blood glucose levels, and is a potential target for designing drugs for the treatment of diabetes [16]. At present, the research of α-glucosidase inhibitors is based on this target. It can delay the decomposition and digestion of complex carbohydrates and disaccharides by inhibiting the activity of α-glucosidase that decomposes oligosaccharides into monosaccharides [17]. Thus, delaying the absorption of glucose in the intestinal cavity and lowering the postprandial blood glucose. Chronic hyperglycemia caused by insulin resistance or insufficient insulin secretion is the characteristic of T2DM [18]. Therefore, insulin resistance plays a key role in the pathogenesis of T2DM and improving insulin resistance is an effective way to improve T2DM. In addition to insulin resistance, the increase in endogenous glucagon will promote gluconeogenesis, enhance glycogen degradation, and inhibit glycogen synthesis to stimulate liver glucose production [19]. This may be a goal of reducing blood glucose and treating diabetes. On the other hand, repairing the damaged hepatic glycogen structure is also a method to improve the abnormal hepatic glycometabolism in diabetes [20]. In addition, carbohydrate digestion and glucose absorption also play an important role in maintaining glucose homeostasis. The digestive process is affected by the activities of a variety of key digestive enzymes, while the absorption process is closely related to the expression level of glucose transporters, intestinal epithelial cell permeability, intestinal barrier function, and other factors [21,22]. Differentiated Caco-2 cells have the morphological and functional characteristics of small intestinal cells, showing brush, such as borders, tight junctions, and uptake of transporters. These transporters regulate the permeability of drugs from intestinal cavity to systemic circulation and are closely related to intestinal permeability and epithelial barrier function [23]. In recent years, there are few reports on the effects of 6-shogaol on glucose uptake and glucose transporters in the studies on its hypoglycemic efficacy. In this study, we explored the effect of 6-shogaol on the activity of α-glucosidase, glucose uptake transport of Caco-2 cells, and the protection of intestinal barrier function, thus revealing the hypoglycemic mechanism. It is expected to provide a valuable reference for the development of dried ginger bioactive substances as hypoglycemic agents.

## 2. Materials and Methods

### 2.1. Materials and Chemicals

Caco-2 cells were purchased from the national experimental cell resource sharing service platform (Beijing, China). The 6-shogaol and α-glucosidase were purchased from Shanghai Yuanye Bio-Technology Co., Ltd. (Shanghai, China). The glucose test kit was purchased from Nanjing Jiancheng Bioengineering Institute (Nanjing, China). Antibodies for Occludin, GLUT2 and Claudin-1 were purchased from Abcam Biotechnology (Cambridge, UK). ZO-1 rabbit antibody and SGLT1 rabbit antibody were obtained from Proteintech Group (Chicago, IL, USA) and ImmunoWay Biotechnology (Plano, TX, USA), respectively. 

### 2.2. Molecular Docking

The structure file of 6-shogaol (PubChem CID:5281794) was prepared from pubChem database. The prepared 6-shogaol file and the three-dimensional crystal structure of α-glucosidase (PDB ID:3W37) from the PDB database were used. The structures were modelled in Discovery Studio 2016, water molecules were removed, hydrogen atoms were added, and the structures were docked. After docking, the activity was evaluated according to docking calculation results.

### 2.3. Determination of α-Glucosidase Activity

Using PNPG (4-Nitrophenyl-β-D-glucopyranoside) method, the reaction system was slightly modified according to the method of predecessors [24]. Firstly, 25 µL of 6-shogaol (500, 300, 200, 100, 50, 25, 12.5, 6.25, 0 μg/mL) and acarbose (100 μg/mL) reacted with 50 μL α-glucosidase, respectively, at 37 °C for 15 min. Then, 100 µL PNPG (10 mmol/L) was added for 30 min. Finally, 50 μL Ca_2_CO_3_ (2 mol/L) was added to terminate the reaction, and the absorbance value was measured at 405 nm. Calculations were conducted according to the following formula:

$$\mathrm{Inhibition}\;\mathrm{rate}\;(\%)\;=\;\frac{\left(A_{1}\;-\;A_{2})\;-\;(A_{3}\;-\;A_{4}\right)}{\left(A_{1}\;-\;A_{2}\right)}\;\times \;100\%$$

where *A*_1_ represents the control, *A*_2_ represents the blank, *A*_3_ represents the sample to be tested, and *A*_4_ represents the sample blank.

### 2.4. Cell Cultures

Caco-2 cells were cultured in DMEM high glucose medium with 10% (*v*/*v*) fetal bovine serum added. The cells were incubated at 37 °C and 5% carbon dioxide humidified atmosphere, and then subcultured when 80–90% confluence was reached. When approaching fusion, cells were digested with trypsin, counted, and inoculated onto transwell culture plate, and the culture medium was replaced every two days. After 1 week, the medium was replaced every day until 21 days.

### 2.5. Determination of Cell Metabolic Activity

The cell concentration was adjusted to 1 × 10^4^ cells/well (100 μL) on 96-well plate. After 24 h of culture, the old medium was replaced by 100 μL medium containing different concentrations (0, 5, 10, 20, 40, 60, 100 μg/mL) of 6-shogaol. After continuous cultivation for 24 h, 10 μL CCK8 was added to each well, and the absorbance was measured at 450 nm after incubation for 2 hours. Calculations were conducted according to the following formula:

$$\mathrm{Cell}\;\mathrm{Metabolic}\;\mathrm{Activity}\;(\%)\;=\;\frac{A_{1}\;-\;A_{0}}{A_{2}\;-\;A_{0}}\;\times \;100\%$$

where *A*_0_ represents the blank group; *A*_1_ represents the sample to be tested; *A*_2_ represents the control.

### 2.6. Measurement of Transmembrane Resistance Value TEER

The cell concentration was adjusted to 2.5 × 10^5^ cells/well (0.5 mL was added to upper chamber, 1.5 mL was added to the basal chamber) on 6-well plate, and each plate was set with a cell-free well as a control. The culture medium was changed every 2 days and changed every day after one week for 21 days. During this period, the Millicell^®^ ERS-2 volt-ohm meter (EMD Millipore Corporation, USA) was used to monitor the transmembrane electrical resistance (TEER) of each well and check the integrity of cells for three consecutive weeks. Calculations were conducted according to the following formula:

TEER value (Ω∙cm^2^)= (R_1_ − R_0_) × 1.1 cm^2^ (effective area of 12-well plate)

where R_1_ represents the resistance value of each well and *A*_0_ represents the resistance value of the blank group.

### 2.7. Determination of Glucose Content

The cell concentration was adjusted to 2.5 × 10^5^ cells/well (0.5 mL was added to upper chamber, 1.5 mL was added to the basal chamber) on 6-well plate. After the 21-day process, it was replaced with serum-free DMEM culture medium for starvation overnight. The cells were divided into five groups: blank group (without cells), control group (with cells; without 6-shogaol), and dose group (with cells; 5, 10, 20 μg/mL of 6-shogaol). The content of glucose in cell culture medium was detected at 12 h and 24 h, according to the manufacturer’s instructions of the glucose kit.

### 2.8. Western Blot Analysis

After cells in each group were treated with 6-shogaol of different concentrations for 24 h, the culture medium was discarded. After PBS washing, the prepared lysis solution (mammalian protein extraction kit solution and protein inhibitor cocktail solution in a proportion of 1:99) was added and placed on ice for complete lysis. Then, cells and lysis solution were transferred to centrifuge tubes to separate supernatant. The protein concentration was determined with BSA kit and adjusted to be consistent. Protein sample and 5×SDS-PAGE loading buffer was mixed and boiled, the target protein was separated by SDS gel electrophoresis with a sample loading of 40 μg/lane. It was transferred to the membrane, sealed, incubated with antibodies, and colored under the illumination instrument [25].

### 2.9. Statistical Analysis

GraphPad Prism 9.1.0 software was used to process and analyze data, expressed in mean ± SEM. One-way analysis of variance was used for significant differences, *p* < 0.05 or *p* < 0.01, and it was considered to be significantly different or extremely significantly different from the control group.

## 3. Results

### 3.1. Molecular Interaction

**Figure 1:**
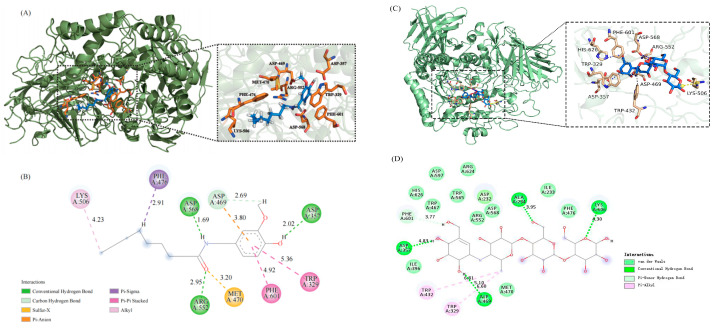
Molecular models of 6-shogaol and acarbose binding to α-glucosidase protein: (**A**) 3D model of 6-shogaol and α-glucosidase (PDBID:3A4A) crystal structure docking. (**B**) 2D model of 6-shogaol and α-glucosidase docking. (**C**) 3D model of acarbose and α-glucosidase (PDBID:3A4A) crystal structure docking. (**D**) 2D model of acarbose and α-glucosidase docking.

Molecular docking technology was beneficial to the discovery of new α-glucosidase inhibitors [26]. Discovery Studio 2016 (DS2016) was used for molecular simulation and the binding sites and forces were shown in Figure 1. Both 6-shogaol and acarbose can effectively bind to the active pocket of α-glucosidase protein, and their binding energies are −6.24 and −7.20 (kcal/mol), respectively, indicating that they have high affinity with α-glucosidase. The 6-shogaol interacts with α-glucosidase under the combined action of conventional hydrogen bond, carbon hydrogen bond, pi-sigma, pi-pi stacked, and other forces. Among them, the conventional hydrogen bonds were formed with the amino acid residues ASP568 (1.69 Å), ASP357 (2.02 Å), and ARG552 (2.95 Å), and the bond length formed with ASP568 (1.69 Å) was the shortest and the binding was the closest. Carbon hydrogen bond was formed with ASP469 (2.69 Å). Sulfur-X was formed with MET470 (3.20 Å). Pi-anion was formed with ASP469 (3.80 Å). Pi-sigma were formed with PHE476 (2.91 Å). Pi-stacked were formed with PHE601 (4.92 Å) and TRP329 (5.36 Å). Forming alkyl was formed with LYS506 (4.23 Å). Acarbose interacts with α-glucosidase under the combined action of van der Waals force, conventional hydrogen bond, pi-donor hydrogen bond, and pi-alkyl. The van der Waals forces were formed with the amino acid residues HIS626, TRP467, ASP597, TRP565, ARG624, ASP568, ARG552, ILE233, MET470, and PHE476. Conventional hydrogen bonds were formed with ASP357 (4.83 Å), ASP469 (5.10 Å), ALA234 (3.95 Å), and LYS506 (4.30 Å). Pi-donor hydrogen bond was formed with PHE601. The pi-alkyl bonds were formed with TRP432 (6.81 Å) and TRP432 (6.60 Å).

### 3.2. Inhibitory Effect on ɑ-Glucosidase

**Figure 2:**
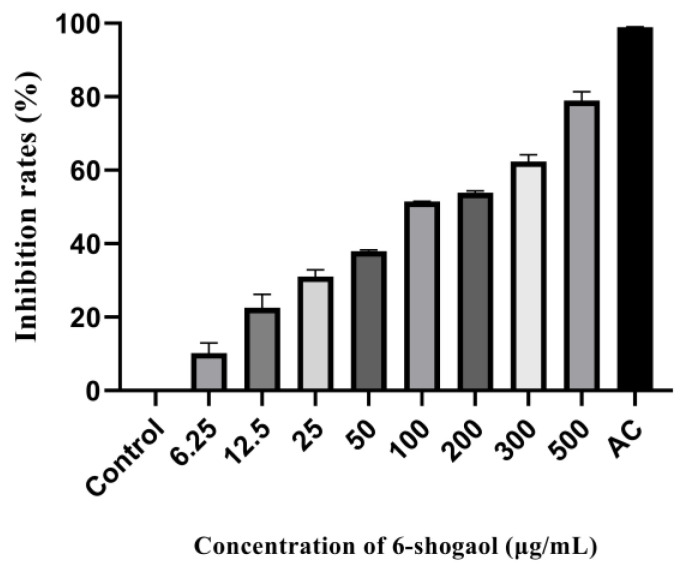
Inhibition of α-glucosidase activity by 6-shogaol.

Preliminarily, we explored the inhibition of 6-shogaol on α-glucosidase. It can be seen from Figure 2 that the inhibition rate of 6-shogaol and acarbose groups was significantly increased compared with the control group. In the positive control group, the inhibition rate of acarbose with the concentration of 100 μg/mL was 98.94%. The activity of α-glucosidase was inhibited by 6-shogaol at different doses. When the concentration of 6-shogaol was 500 μg/mL, the inhibition rate was 78.96%. The results showed that 6-shogaol inhibited the activity of α-glucosidase in a dose-dependent manner. As a typical glucosidase inhibitor, acarbose has shown strong inhibition ability.

### 3.3. Caco-2 Cell Transmembrane Resistance

**Figure 3:**
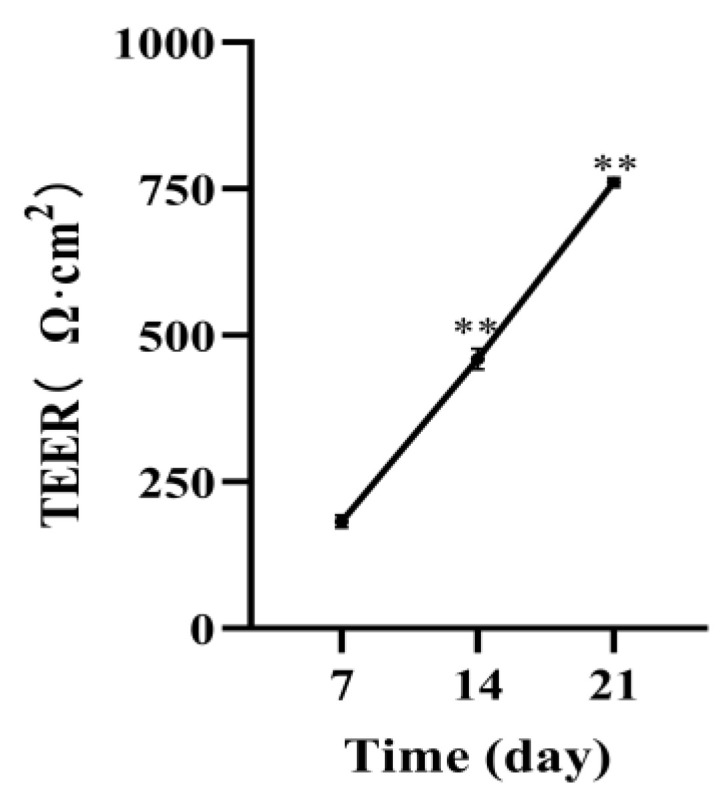
Change in TEER value of Caco-2 cells within 21 days. * represents significance, as compared with the TEER value on the 7th day (** *p* < 0.01).

To evaluate the monolayer integrity of differentiated Caco-2 cells, we measured the TEER within three weeks. As shown in Figure 3, with the extension of cell culture time, the monolayer transmembrane resistance of cells continued to increase every week. It has been > 500 Ω∙cm^2^ at 21 days, indicating that Caco-2 cells have formed a dense monolayer structure.

### 3.4. Effect on the Activity of Caco-2 Cells

**Figure 4:**
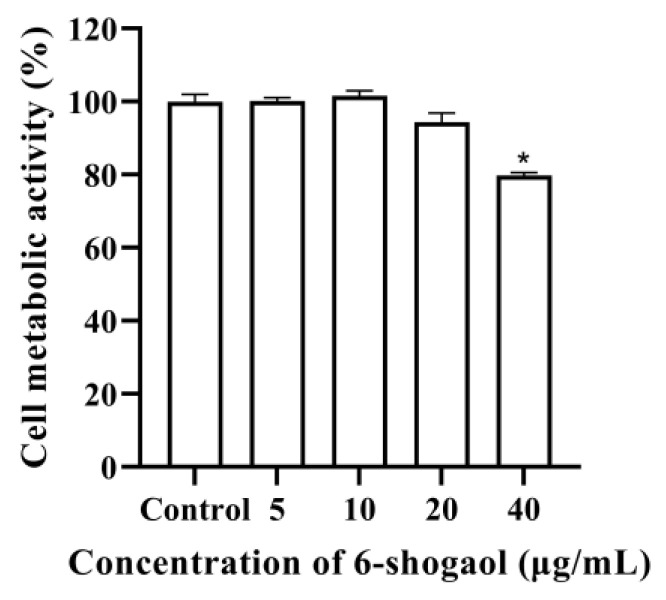
Effect of 6-shogaol on the metabolic activity of Caco-2 cells. * represents significance, as compared to control group (* *p* < 0.05).

CCK8 method was used to detect the effect of 6-shogaol on the metabolic activity of Caco-2 cells. It can be seen from Figure 4 that the activity of Caco-2 cells was significantly reduced when the concentration of 6-shogaol was 40 μg/mL, while there was no significant difference between the control group and the concentration of 5, 10, and 20 μg/mL. Therefore, it is considered that 6-shogaol has no toxic effect on cells when its concentration was 5, 10, and 20 μg/mL, and subsequent experiments were carried out.

### 3.5. Effect on Glucose Consumption of Caco-2 Cells

**Figure 5:**
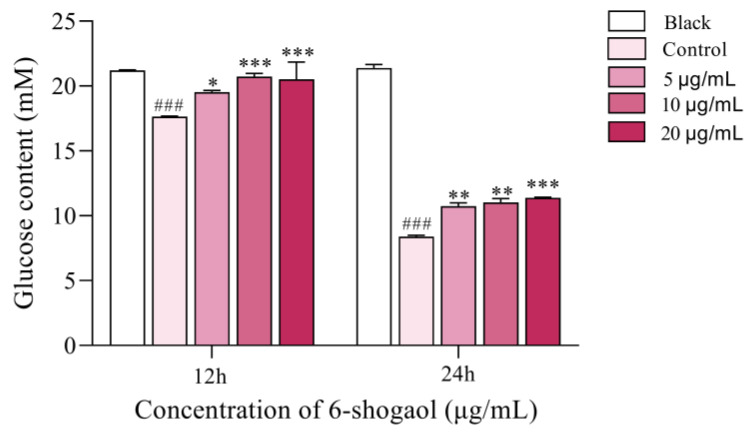
Effect of 6-shogaol on glucose consumption in Caco-2 cells. # compared with blank (### *p* < 0.001), * represents significance, as compared to control group (*** *p* < 0.001, ** *p* < 0.01, * *p* < 0.05).

Figure 5 shows the results of glucose content in culture medium after intervention of 6-shogaol for 12 h and 24 h. It can be seen that, with the extension of time, the residual glucose in the control group and 6-shogaol intervention group decreased significantly compared with the blank group, which is due to the consumption of glucose intake energy in the process of cell growth. Compared with the control group, the glucose content of 6-shogaol at different concentrations increased after intervention, indicating that 6-shogaol inhibited the glucose uptake of cells.

### 3.6. Effect on the Expression of Related Proteins in Caco-2 Cells

**Figure 6:**
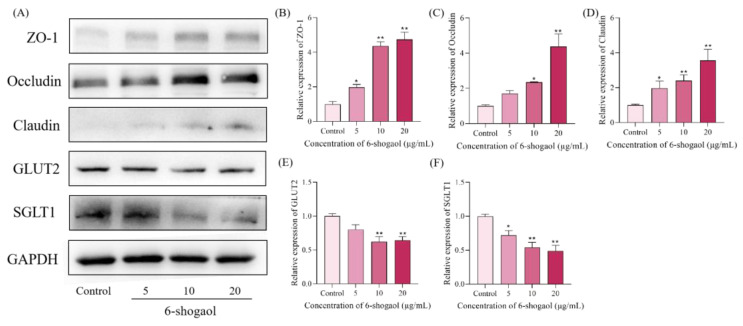
Effect of 6-shogaol on the expression of related proteins in Caco-2 cells. (**A**) The expressions of ZO-1, Occludin, Claudin, GLUT2, and SGLT1 were analyzed by Western blot. (**B**–**F**) The quantitative analysis of protein expressions in Caco-2 cells. * represents significance, as compared to control cells (** *p* < 0.01, * *p* < 0.05).

Western blot results were shown in Figure 6. The expression of related proteins was regulated by 6-shogaol in varying degrees. GLUT2 and SGLT1 are key proteins in the glucose transport process, and ZO-1, Occludin and Claudin are tight junction proteins, which are related to the integrity and permeability of the intestinal barrier. It can be seen from Figure 6 that due to the intervention of 6-shogaol, the relative expression of ZO-1, Occludin, and Claudin proteins was significantly increased, and the relative expression of GLUT2 and SGLT1 proteins was decreased, especially at high concentrations. This suggests that 6-shogaol can inhibit glucose transport and protect the intestinal barrier.

## 4. Discussion

The monitoring and treatment of T2DM has been a hot topic of research and concern for decades. The American Diabetes Association predicts that diabetes patients will increase to 10.2% (578 million) by 2030 and 10.9% (700 million) by 2045, which indicates that diabetes has become a global epidemic health problem [27]. The main manifestation of T2DM is the increase in blood glucose. The effective control of postprandial blood glucose increase is an effective measure to reduce blood glucose. When the body ingests food, carbohydrates are digested into glucose under the action of digestive enzymes in the gastrointestinal tract. α-glucosidase belongs to carbohydrate digestion enzyme and is the rate-limiting enzyme for glucose production. Inhibiting its activity can reduce the production of glucose. Furthermore, glucose is absorbed by epithelial cells through the brush border membrane by the intestinal tract depending on specific glucose cotransporters. The glucose enters into the blood circulation, causing the blood glucose to rise [28]. Therefore, the inhibition of carbohydrate digestion and glucose absorption and transport in the intestine is an effective way to reduce blood glucose. Therefore, we considered the influence of 6-shogaol on the process of glucose digestion, absorption, and transport in order to clarify the mechanism of 6-shogaol’s hypoglycemic effect.

α-glucosidase is a key enzyme responsible for digesting dietary carbohydrates into glucose. It hydrolyzes glycosidic bonds in various sugar compounds by means of nuclear or exonuclear cleavage to produce monosaccharides, oligosaccharides, or glycosaminoglycans [29]. Hence, reducing α-glucosidase activity could delay glucose release and further absorption. In this study, the inhibitory effect of 6-shogaol on α-glucosidase was predicted by molecular docking technology. Molecular docking is a method to predict the position and affinity of ligands at receptor binding sites, which is often used in drug design research [30]. It is noteworthy that it has been widely used in food science in recent years, such as in the study of enzyme activity and substrate [31]. The interaction process between the receptor and ligand includes hydrogen bonds, electrostatic interaction, van der Waals force, hydrophobic interaction, etc. [32]. The strength of these interactions is crucial to evaluate the affinity between the receptor and ligand. In the study of the inhibitory effect of pepper plant chemical components on α-glucosidase, luteolin showed a strong inhibitory effect on α-glucosidase, which may be related to the formation of hydrogen bonds and hydrophobic interaction of the key amino acid residues of α-glucosidase [33]. In a study on the inhibitory effect of traditional Chinese medicine ingredients on α-glucosidase, supramolecular docking was used to simulate and clarify its mechanism [29]. The results showed that its inhibitory effect originated from bonding with the residue of α-glucosidase. Generally speaking, the lower the energy required for the combination of the ligand receptor, the easier it is to dock. When the binding energy is less than 0 kcal/mol, it is considered to be able to bind spontaneously [34]. Marilisa et al. predicted the high affinity of chromogenic acids to α-glucosidase by molecular docking, obtained the negative binding energy, and pointed out that the predicted enzyme-substrate binding was spontaneous and the affinity for α-glucosidase was higher than that of acarbose [35]. In this study, we predicted the inhibition of α-glucosidase by 6-shogaol through molecular docking technology. The 6-Shogaol and α-glucosidase are docked through conventional hydrogen bond, carbon hydrogen bond, pi-sigma, pi-pi stacked, and other forces. Additionally, the docking results of acarbose and α-glucosidase showed that the van der Waals force, conventional hydrogen bond, pi-donor hydrogen bond, and pi-alkyl were formed between them. The binding energy of 6-shogaol and acarbose with α-glucosidase protein are −6.24 and −7.20 kcal/mol, which means it is considered that they can spontaneously bind with α-glucosidase and have high binding affinity. This result suggests that 6-shogaol has potential hypoglycemic activity. Compared with 6-shogaol, the lower binding energy of acarbose and α-glucosidase may be related to the formation of more hydrogen bonds and van der Waals force. 

Furthermore, the enzyme activity experiment in vitro was carried out to verify the results of molecular docking to confirm the inhibitory effect of 6-shogaol on α-glucosidase. A series of concentration gradients were selected for the enzyme activity test to show the inhibition of 6-shogaol more clearly on α-glucosidase and its dose-dependent effect. Our previous study showed that the 6-shogaol content in ginger reached 531 µg/g [36], and clinical study showed that the toxicity of 2 g ginger per day on the human body was negligible. Therefore, we suggest 500 µg/g as the maximum concentration. The results showed that 6-shogaol had a strong inhibitory effect on α-glucosidase in a dose-dependent manner [37]. Differentiated Caco-2 cells are mature human intestinal mucosal models, which can differentiate into brush border membranes and basolateral membranes and express a variety of enzymes and nutrient transporters in vitro [38]. TEER value indicates the resistance of the cell monolayer, which can be used to verify the integrity of the barrier. When TEER value ≥ 500 Ω, it indicates that the cells have formed a dense monolayer structure [21]. In our study, the TEER value of Caco-2 cells on the 21st day of differentiation in transwell culture plate was more than 500 Ω, indicating that a monolayer structure has been formed, which is similar to the previous results [28]. Under our test conditions, the CCK8 test showed that 5, 10, and 20 μg/mL of 6-shogaol had no toxic effect on cells, so the maximum concentration used in the test was 20 μg/mL. Furthermore, we measured the glucose content in the cell culture medium of each group and found that the glucose content increased after 6-shogaol intervention, indicating that 6-shogaol inhibited the glucose uptake of cells. Similarly, previous studies have shown that anthocyanins, coumaric acid, and quercetin can inhibit the glucose uptake of Caco-2 cells [39].

Caco-2 cells after differentiation are similar to intestinal absorption cells in morphology, with microvilli and tightly connected cell monolayers and related transporters [40]. The transport of glucose through intestinal brush border membrane plays a key role in metabolic regulation. Glucose is mainly transported by sodium-dependent glucose transporter (SGLT1) and facilitated-transporter glucose transporter (GLUT2) [41]. When the glucose concentration in the intestine cavity is low, its transport mainly depends on the active transport of SGLT1 to transfer the glucose in the intestine cavity to the epithelial cells. However, when the concentration is high, glucose is mainly transported into the blood by GLUT2 [28]. Studies have shown that flavonoids can reduce the glucose uptake of Caco-2 cells by reducing the expression of glucose transporter gene and inhibiting the binding sites of SGLT1 and GLUT2 [42]. In this study, 6-shogaol intervention also reduced the relative expression of Caco-2 cells transporters SGLT1 and GLUT2, especially at high concentrations. This suggests that 6-shogaol may reduce the glucose uptake and utilization of Caco-2 cells by inhibiting the expression of related transporters. Occludin, Claudin, and ZO-1 play a crucial role in establishing cell–cell contact and maintaining cell bypass permeability. In the study on the enhancement of Caco-2 cells barrier by flavonoids in black ginger, it was shown that the increase in the expression of Occludin and Claudin-1 proteins in cells enhanced the integrity of the barrier, increased the trans epithelial resistance, and decreased the permeability of glucan [43]. In a study based on the Caco-2/HT29 coculture model, attempting to evaluate the effect of indole-3-propionic acid on intestinal barrier function, it was found that the increase in trans epithelial resistance was consistent with the increase in tight junction proteins (Claudin-1, Occludin, and ZO-1) [44]. Similarly, in our study, the relative expression of the tight junction proteins Occludin, Claudin, and ZO-1 was up-regulated in the 6-shogaol group. It shows that 6-shogaol has a certain protective effect on intestinal barrier function.

## 5. Conclusions

We preliminarily predicted the inhibition of α-glucosidase by 6-shogaol using molecular docking technology and verified it based on the enzyme activity experiment, proving that 6-shogaol can inhibit the activity of α-glucosidase. Furthermore, based on the differentiated Caco-2 cell model, the effects of 6-shogaol on glucose absorption and transport and intestinal barrier protection were investigated. The results showed that 6-shogaol could reduce the glucose uptake of cells, inhibit the glucose transport by down regulating the expression of glucose transporters (SGLT1 and GLUT2), and enhance the intestinal barrier function by up regulating the expression of tight junction proteins (Claudin-1, Occludin, and ZO-1). This study provides a new way for 6-shogaol to treat T2DM and provides theoretical support for the development and utilization of functional products of dried ginger.

## Data Availability

The data presented in this study are available on request from the corresponding author.
